# Isolation of a 97-kb Minimal Essential MHC B Locus from a New Reverse-4D BAC Library of the Golden Pheasant

**DOI:** 10.1371/journal.pone.0032154

**Published:** 2012-03-05

**Authors:** Qing Ye, Ke He, Shao-Ying Wu, Qiu-Hong Wan

**Affiliations:** The Key Laboratory of Conservation Biology for Endangered Wildlife of the Ministry of Education and State Conservation Center for Gene Resources of Endangered Wildlife, College of Life Sciences, Zhejiang University, Hangzhou, China; University of South Florida College of Medicine, United States of America

## Abstract

The bacterial artificial chromosome (BAC) system is widely used in isolation of large genomic fragments of interest. Construction of a routine BAC library requires several months for picking clones and arraying BACs into superpools in order to employ 4D-PCR to screen positive BACs, which might be time-consuming and laborious. The major histocompatibility complex (MHC) is a cluster of genes involved in the vertebrate immune system, and the classical avian MHC-B locus is a minimal essential one, occupying a 100-kb genomic region. In this study, we constructed a more effective reverse-4D BAC library for the golden pheasant, which first creates sub-libraries and then only picks clones of positive sub-libraries, and identified several MHC clones within thirty days. The full sequencing of a 97-kb reverse-4D BAC demonstrated that the golden pheasant MHC-B locus contained 20 genes and showed good synteny with that of the chicken. The notable differences between these two species were the numbers of class II B loci and NK genes and the inversions of the TAPBP gene and the TAP1-TAP2 region. Furthermore, the inverse TAP2-TAP1 was unique in the golden pheasant in comparison with that of chicken, turkey, and quail. The newly defined genomic structure of the golden pheasant MHC will give an insight into the evolutionary history of the avian MHC.

## Introduction

The bacterial artificial chromosome (BAC) is a type of plasmid vectors permitting stable propagation of cloned inserts greater than 100 kb [1]. The ability of BAC vector to accommodate such large inserts makes it a powerful tool of genome biology studies [2]. The BAC library is widely constructed for comparative genomics of some large-size genomic regions of interest, such as major histocompatibility complex (MHC) region [3], [4].

The MHC consists of a number of multi-gene family members involving in the immune responses of vertebrates [5]. Due to its important role in immunity and its exceptionally high level of genetic variation, the MHC has attracted considerable attention from many different fields of biological researches, especially for mammals [6]. The mammalian MHC region always occupies more than half a million kilobases in length, as revealed in human [7], cow [8], pig [9], dog [10] and giant panda [11], which were determined by constructing BAC genomic library and physical map. The intact avian MHC genomic data were available from chicken [12], quail [13] and turkey [3] and all of them showed that the *Galliform* possessed a minimal essential MHC genomic structure spanning about one hundred kiolobases, which is so small that one BAC is enough to hold.

The chicken MHC is a pioneer and best-studied study of birds [12], [14]. The first map of the MHC-B region of the chicken and its recently extended map both have well defined genes in chicken MHC-B [12], [15]. Although MHC sequence variation has been studied in a large number of other bird species [16]–[21], most of these studies have only characterized a small part of one or a few loci rather than the large-scale genomic structure and organization of the MHC genes. Currently, the detailed information on large-scale bird MHC-B organization is available in two other *Galliform* birds, turkey [3] and quail [13]; one is in near-perfect synteny with chicken, and the other is of higher degree of gene duplication, longer introns, and intergenic distances. According to the recently study of zebra finch MHC, it is a complex one involving gene duplication and fragmentation [22]. Consequently, it needs more *Galliform* species to be studied to confirm the minimal essential structure in *Galliform*.

Golden pheasant (*Chrysolophus pictus*) is a national second-class protected species endemic to China and is listed as near threatened (NT) in China Species Red List [23]. Because of its beautiful feathers and especial function in traditional Chinese medicine, this species has declined dramatically in wild. At present, genetic studies of this endangered pheasant are limited to diversity surveys using microsatellites and mitochondrial DNA [24], [25] and there is no reports of genomic library construction and *Chrysolophus pictus* MHC (*Chpi-MHC*) determination. As an important functional marker system surveying adaptive evolutionary history, the *Chpi-MHC* analysis should be given priority in conservation biology studies of this bird in order to protect it more efficiently.

Generally, the BAC library is encompassed in numerous 384-well or 96-well plates, the number of which decides the genomic coverage of the library. Since constructing a BAC genomic library requires high cost and considerable expertise [1], some researchers improved genomic library construction methods in order to speed up the process. Different kinds of BAC libraries were thus built including chromosome-specific or chromosome arm-specific [26], gene-enriched [27] and non gridded genomic libraries [28]. However, the specific or enriched libraries just stored a partial genome in BAC clones, while the non gridded library had no backup ones and consumed the library gradually. Consequently, it is essential to bring forward a new method incorporating convenience of operation and integrity of library. Here, we developed a new method to construct BAC library, which was characterized by (1) the division of cell cultures into sub-libraries followed by the backuping of sub-libraries and (2) the use of two-round PCR in both screening positive sub-libraries and achieving the target BACs. We successfully identified the BAC clones containing MHC genes in the golden pheasant in a short period. Hence, this study not only provides a convenient and inexpensive method to construct library but also gives an insight into the evolutionary history of the avian MHC.

## Results

### Overview of the new reverse-4D method

**Figure 1:**
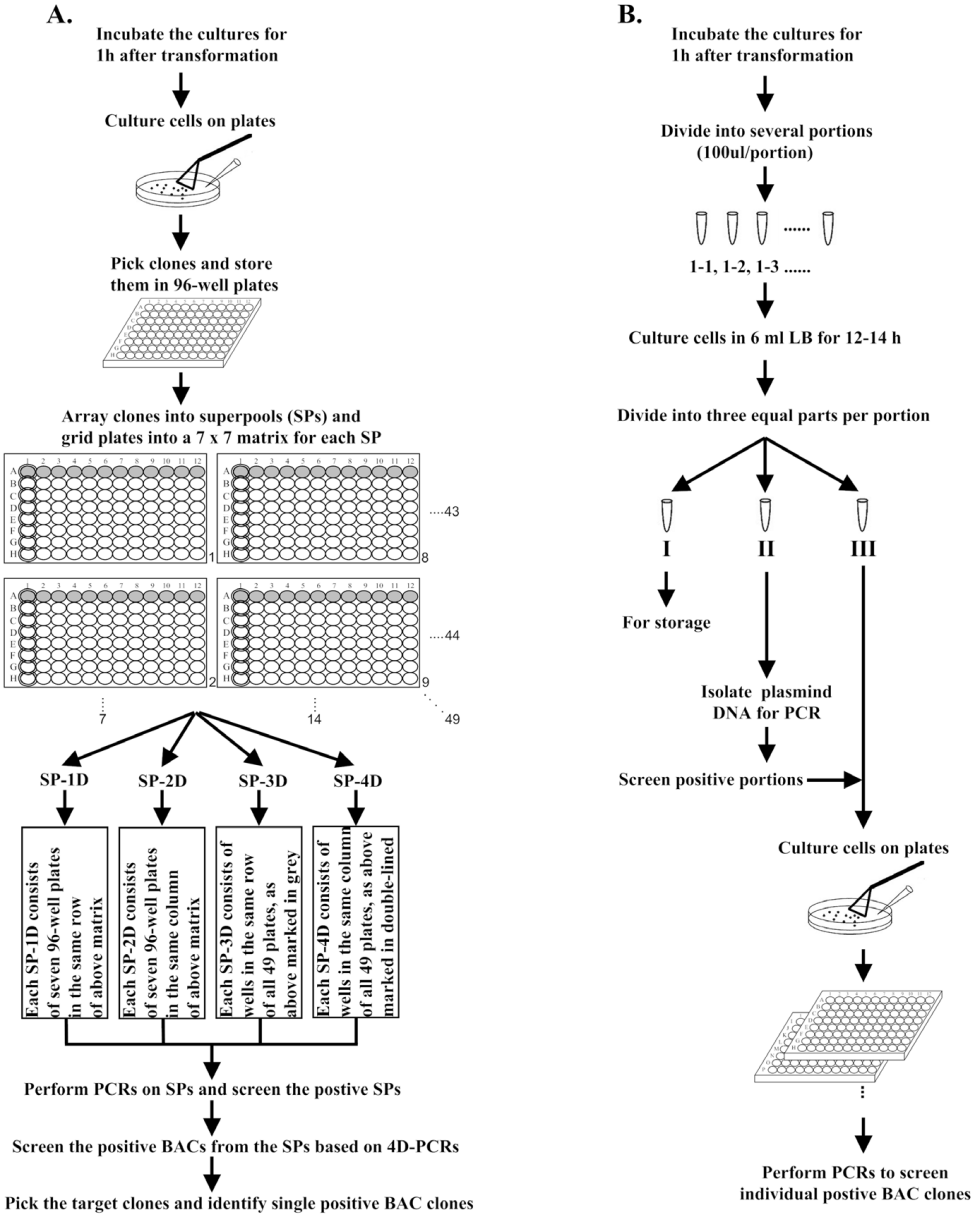
Schematic representation of the process of (A) 4D-PCR method; and (B) Reverse-4D method.

Construction of a traditional BAC genomic library requires the first picking of clones, the second arraying of clones into superpools (SPs), and the final screening of positive clones using 4D-PCR method (Fig. 1A). In this study, we developed a new method, which involved the first division of sub-libraries, the second preparing of backup ones, the third screening of positive sub-libraries and the final achievement of target BACs (Fig. 1B). In this new route of library construction, picking clones was transferred from the first step of routine 4D-based library to the last step of this new library while creating sub-libraries or superpools was changed from conventional second part to the first part in this study (Fig. 1B). Therefore, we generated a genomic BAC library in reverse order to the traditional 4D-based way and thus named it reverse-4D method.

### The number of clones and genomic coverage of the BAC library

**Figure 2:**
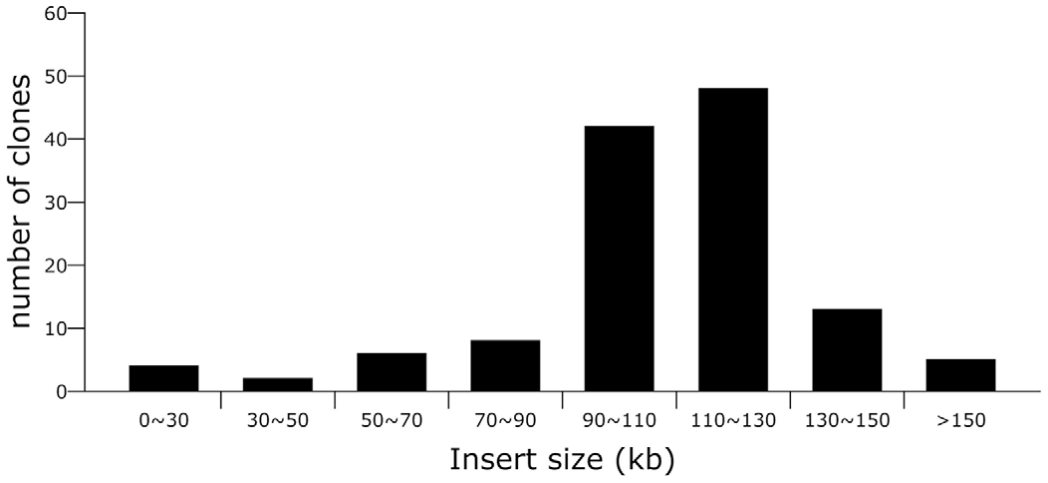
Distribution of insert size of 128 randomly selected clones.

A mini routine BAC library was first constructed to evaluate the average size of inserts, which corresponds to a sub-library in reverse-4D library. The 100-µl cell cultures were grown on 15 10-cm plates and the number of clones ranged from 600 to 1400, averaging 800 per plate. The reverse-4D library of the golden pheasant contained 112 sub-libraries from 4 different ligations and so it comprised about 89600 recombinant clones. A total of 128 randomly selected clones obtained 4 empty ones and the remaining 124 BACs hold inserts 35.6–170.9 kb in size, thus giving the average size as 106.87 kb (Fig. 2) and the empty vector rate as 3.125%. According to the 1.25 C-value of common pheasant and domestic chicken (http://www.genomesize.com/), the coverage of the library was estimated to be 7.421 genome equivalents. The probability of gaining any single copy gene from the library was about 99.95% as calculated by the formula N = ln(1−P)/ln(1−I/GS) [29].

### Gene content and genomic characteristics of the B locus

**Figure 3:**
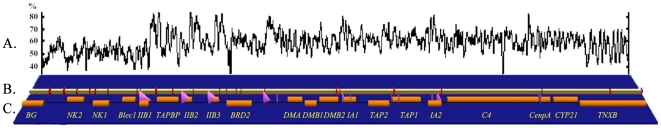
Gene organization of the 97 kb (97,476 bp) *Chpi-MHC-B* (JQ440366). A, Plot of GC content in overlapping 200 bp windows. B, Locations of repeat sequences, as shown in red. C, Genes and CpG islands predicted within *Chpi-MHC-B*. The orange columns flanking the lines indicate genes, of which the upper and lower ones represent forward and reverse orientations, respectively. The pink triangles depict locations of CpG islands.

The genomic insert fragment of No. 9-6-S2 BAC clone showed 97 kb in length (JQ440366) and contained the majority of the homologous genes in the MHC-B region (Fig. 3). A total of 20 genes were identified in the 97 kb region, corresponding to one gene per 4.6 kb (Fig. 3C). The gene density was almost the same as that in other *Galliformes* (chicken, quail and turkey) [3], [12], [13], but four times higher than that in the HLA region [7]. The genes involved partial BG-like, Blec1, two NKs (in order to distinguish gene duplication, the gene were named NK instead of Blec), three MHC class II B loci, TAPBP, BRD2, DMA, two DMBs, two MHC class I loci, TAP1, TAP2, C4, CenpA, CYP21 and partial TNXB (Fig. 3C). As for the complex region containing three IIB loci, the relationship and location of IIB, TAPBP, TAPs and their adjacent genes were verified by five different individuals, indicating that the genomic structure of IIB1-TAPBP-IIB2-IIB3-BRD, and DMB2-IA1-TAP2-TAP1-IA2-C4 were reliable.

The overall GC content of pheasant MHC-B region was 58.46% (Fig. 3A). Compared with 59.27%, 58.0% and 54.6% of chicken, turkey and quail, respectively, it was relatively consistent. The IIB1-TAPBP-IIB2-IIB3 and class I regions both presented an obviously higher GC content than other parts (nearly 65%) (Fig. 3A), suggesting their distinctive function in the MHC-B locus.

Repetitive elements was characteristic in species and could trigger duplication events [11]. The golden pheasant repeats were analyzed to provide an insight into genomic evolution of its MHC-B locus. Several types of repetitive elements were detected, including 5 CR1 repeats and 39 STRs (Fig. 3B). The frequency of SSRs was 1 every 2.64 kb, which was close to chicken and turkey (1 per 2.09 kb and 1 per 2.64 kb) [3], [12]. No tRNA sequences were identified in this region, consistent with corresponding parts of chicken and turkey. The CpG islands were conserved in nine regions (Fig. 3C), which appeared in the same positions as that in chicken and turkey [3], [12]. Nonetheless, the quail had more CpG islands than other three birds, probably reflecting species characteristics [13].

### Genomic comparison and phylogenetic analysis

The MHC genes of the golden pheasant were homologous to the chicken, turkey, and quail, showing 91.4%, 93.2% and 88.4% nucleotide identities and 87.5%, 89.8% and 82.8% amino acid similarities, respectively (Table 1). Collectively, the comparison of homologous genes indicated the golden pheasant had higher homology with turkey than with chicken and quail. As for the separate genes, the ratios of *d*_N_/*d*_S_ across four species revealed that the antigen-presentation genes (IIB and IA) underwent positive selection [30], especially on their antigen-presentation domains (all >1; Table 1).

**Table 1:** Genes identified in the golden pheasant MHC-B and comparisons with homologous ones from chicken (AB268588), turkey (DQ993255) and quail. (AB078884).

		Golden pheasant			Chicken			Turkey		Quail		
Location	Gene name	Orientation of genes	Number of exons	Base pair	Amino acid	Nucleotide identity	Amino acid identity	Nucleotide identity	Amino acid identity	Nucleotide identity	Amino acid identity	*d*_N_/*d*_S_ a
<1–3333	BG	−	Partial									
6770–9316	NK2	+	6	678	225	0.851	**0.716**	0.892	0.813	0.891	0.822	0.917
11044–13537	NK1	−	6	681	226	0.877	0.751	0.913	0.845	0.893	0.805	0.982
15384–17415	Blec1	+	5	567	188	0.942	0.926	0.951	0.941	0.924	0.846	0.378
18280–19678	IIB1	−	6	792	263	0.917	0.856	0.922	0.886	0.874	0.806	*1.009/1.037* b
20805–24134	TAPBP	+	8	1293	430	0.924	0.912	0.953	0.944	0.891	0.879	0.300
24838–26239	IIB2	+	6	792	263	/	/	0.932	0.891	0.885	0.817	*1.140/1.037* b
29026–30437	IIB3	+	6	768	255	0.912	0.829	0.910	0.847	0.867	0.765	*1.213/1.037* b
31795–35563	BRD2	−	11	2202	733	0.959	0.996	0.971	0.996	0.937	0.995	0.015
41243–43537	DMA	+	4	792	263	0.928	0.894	0.955	0.951	0.871	0.814	0.528
43910–45796	DMB1	+	6	738	246	0.912	0.837	0.908	0.846	0.866	0.768	0.696
46423–49190	DMB2	+	6	777	258	0.929	0.926	0.961	0.961	0.861	0.833	0.371
49981–51979	IA1	+	8	1083	349	0.863	0.767	**0.877**	**0.781**	0.848	0.742	0.904/*1.449* b
53847–57081	TAP2	−	9	2007	701	0.924	0.927	0.950	0.950	0.910	0.893	0.256
57624–62037	TAP1	+	10	1674	587	0.934	0.923	0.964	0.959	0.901	0.910	0.256
63132–65148	IA2	−	8	1077	360	**0.845**	0.730	0.894	0.809	**0.836**	**0.719**	0.994/*1.449* b
66230–80353	C4	+	38	5225	1623	0.935	0.937	0.954	0.954	/	/	0.241
80803–82333	CenpA	+	4	401	131	0.965	0.992	/	/	/	/	0.037
82837–86607	CYP21	+	8	1437	478	0.927	0.948	/	/	/	/	0.168
Average	/	/	/	1309	431	0.914	0.875	0.932	0.898	0.884	0.828	

The lowest values in nucleotide and amino acid identities were shown in bold.

a The ratios of *d*_N_/*d*_S_ were calculated across golden pheasant, chicken, turkey and quail, of which the values larger than 1 were indicated in italic.

bThe values calculated from antigen presentation domains of all IIB or IA loci were shown as well.

**Figure 4:**
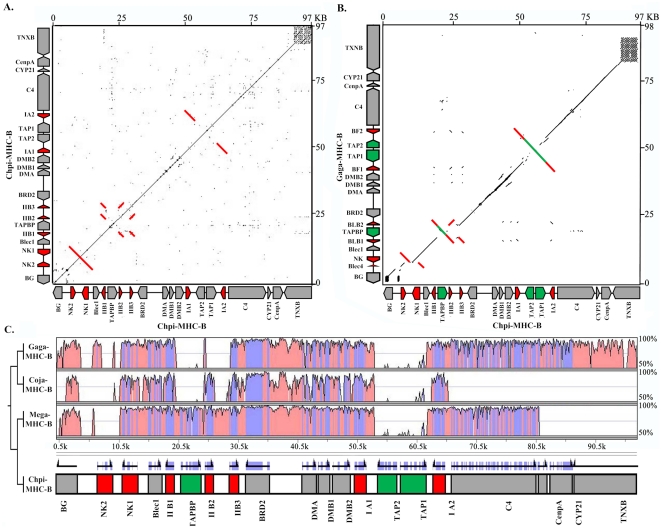
A, A dot plot of *Chpi-MHC-B* itself. B, Inter-species dot plot of the *Chpi-MHC-B* and *Gaga-MHC-B*. C, A VISTA plot of multiple alignments from four avian MHC-B sequences. Percentage of identity plots are displayed by pink blocks and the blue regions indicate locations corresponding to exons. The duplicate blocks detected by dot plot and the corresponding genes inside were indicated in red while the inversed genes relative to chicken were shown in green.

**Figure 5:**
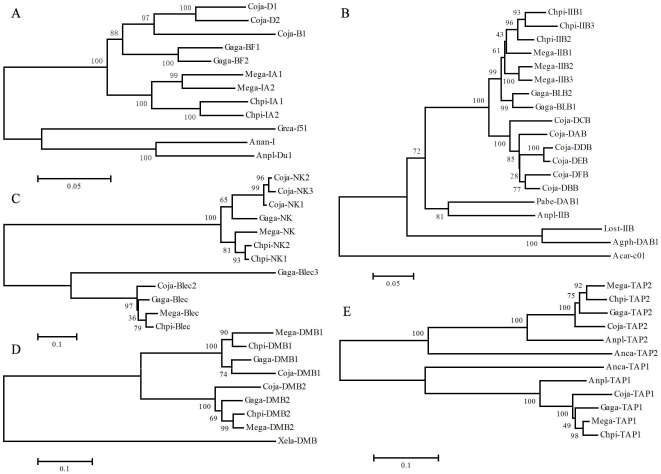
Phylogenetic trees of IA (A), IIB (B), NK-Blec (C), DMB (D) and TAP (E) genes. For the turkey, chicken and quail, the coding regions of genes were extracted from genomic sequences of *Mega-MHC* (DQ993255), *Gaga-MHC* (AB268588) and *Coja-MHC* (AB078884), respectively. The following sequences were also included for tree building: For the IAs: AF033106 (*Grus Canadensis*, *Grca*-f51), AY387652 (*Anser anser*, *Anan*), and AB115241 (*Anas platyrhyncho*s, *Anpl*-Du1); For the IIBs: L42335 (*Lonchura striata*, *Lost*), DQ490139 (*Anas platyrhyncho*, *Anpl*), AJ404372 (*Acrocephalus arundinaceus*, *Acar*-c01), AF170972 (*Agelaius phoeniceus*, *Agph*-DAB1), and FJ588549 (*Pachytila belcheri*, *Pabe*-DAB1); For the DMB: DQ268506 (*Xenopus laevis*, *Xela*); For the TAPs: AY885227 (*Anas platyrhynchos, Anpl*-TAP1 *and Anpl*-TAP2), XM_003230087 (*Anolis carolinensis*, *Anca*-TAP1), XM_003229647 (*Anolis carolinensis*, *Anca*-TAP2). The NK and Blec members of lection superfamily were all from the same genomic sequences of DQ993255, AB268588 and AB07888.

MHC B fragment of gold pheasant has undergone frequent events and evolved two copies of NK gene (NK1 and NK2), three copies of MHC class II classical β gene (IIB1, IIB2 and IIB3), two copies of MHC class II non-classical β gene (DMB1 and DMB2), two copies of TAP gene (TAP1 and TAP2) and two copies of MHC class I gene (IA1 and IA2) (Fig. 3). However, the self-sequence dot plot of the golden pheasant MHC-B locus just revealed the duplication events of the NK, IIB and IA genes and had no evidence of recently duplicating the DMB and TAP genes (Fig. 4A), which suggested DMB and TAP genes duplicated in ancestors. Phylogenetic trees showed that the NK, IIB and IA genes all formed a highly-supportive clustering specific to the golden pheasant (>90% bootstrap values; Fig. 5). On the contrary, phylogenies of the DMB and TAP genes showed that the sequences were grouped according to gene categories rather than species; producing the DMB1 and DMB2 branches and the TAP1 and TAP2 clusters (Fig. 5D–E). As a result, multiple copies of the NK, IIB and IA genes were derived from younger intra-species duplication events, while the duplicated DMBs or TAPs were generated by ancestral triggering.

Dot plot analysis of the MHC-B regions between the chicken and golden pheasant revealed a similar result, namely duplication happened in the genes of NK, IIB and IA (Fig. 4B). Nevertheless, the dot plot discriminated two inversion events of the TAPBP and TAP1-TAP2 regions, respectively (Fig. 4B), which were confirmed by VISTA plot between chicken and pheasant (Fig. 4C). To confirm the inversion, LR PCR was taken to verify the gene location and direction of IIB-TAPBP-IIB, DMB-IA-TAP and TAP-IA-C4 in five individual and 9-6-S2 BAC clone. Except for these duplicated and inversed genes, other regions were in good synteny with those of chicken, turkey and quail (Fig. 4B–C).

The VISTA plots between the golden pheasant and other three birds effectively distinguished some species-specific characteristics (Fig. 4C). The golden pheasant presented a unique TAP2-TAP1 orientation in its MHC-B region. On the contrary, other three birds had an inverse TAP1-TAP2, all resulting in absence of similarity of this region in the VISTA plots (Fig. 4C). Similarly, the golden pheasant and turkey had a forward-orientation TAPBP but the chicken and quail showed a reverse one, leaving blanks at the corresponding plot regions (Fig. 4C). On the other hand, besides inversion, the loss of genes also could produce blanks in VISTA plots. Within these four MHC-B fragments compared, the blanks at the NK and IIB plot regions indicated that only the golden pheasant had two NK genes, one of which became pseudo in chicken (Fig. 4B–C), and that the golden pheasant, turkey and quail all had three IIB genes but the chicken possessed two IIB loci (Fig. 4C). All of these differences together reflect species-specific evolutionary history in the MHC region.

## Discussion

### Reverse-4D method

Construction of a traditional 4D-based genomic library normally first picks clones and then creates superpools (Fig. 1A). Here, we built a reverse-4D library (Fig. 1B) and reduced the time to obtain a positive BAC from original five months to current one month, greatly accelerating the progress of isolating few large genomic fragments or BACs. With the development of next-generation sequencing technology, we have entered the era of fast and inexpensive genome sequencing, which renders limit use to BAC genomic library in genome projects. Nonetheless, genome size of animals and plants is so large that the whole genome fine map leaves many gaps. If one is interested in a certain region interrupted by gaps, we have to employ few BACs to fill gaps between scaffolds. In this case, constructing a reverse-4D BAC library would be faster and more economical. On the other hand, some large, complex and repeat-rich genomic regions are unable to be assembled successfully, as shown in zebra finch MHC [31], of which the classical MHC region was largely absent in the scaffolds. In this case, BAC-based assembly is absolutely necessary. As a result, the reverse-4D library has obvious advantages over traditional 4D-based library for achieving few large genomic fragments several hundred kb in length.

Although the first sub-library division in the reverse-4D method is able to save up to 80% of time to identify the positive clones from a routine BAC library, the second creation of three equal parts might induce some problems due to inadequate blending. For example, there would be no positive individual BAC clones from the part-III in spite of positive present in the corresponding part-II of the same sub-libraries. This means loss of the target clones in subculture or an extremely low percent of the target clones. In order to get rid of the heterogeneity caused by blending, PCR will be taken to recheck whether there are target clones in part-I. Large-scale culture of the corresponding part-I could be adopted to overcome this difficulty on the premise of positive; otherwise this sub-library will be abandoned. Meanwhile, it should be pointed out that homogeneous mixing is of critical importance for dividing a sub-library into three parts.

### Genomic comparison between the golden pheasant and chicken MHC-B loci

The overall MHC-B region of the golden pheasant shows good synteny with that of chicken (Fig. 4B), having a tremendous number of conserved genes (Fig. 4). Nevertheless, the MHC is a hotspot of recombination [32] and thus the *Chpi-MHC* presents frequent events of gene duplication and inversion (Fig. 4).

It was found that the TAPBP and TAP1-TAP2 regions were inversed in the golden pheasant when it was compared with chicken (Fig. 4B). Nonetheless, it seems that the flanking regions of these two segments were inversed too (Fig. 4B). The following evidence supported that the inversion events just happened in the regions of TAPBP and TAP2-TAP1 rather than IIB1-TAPBP-IIB2 and IA1-TAP2-TAP1-IA2. (1) The phylogenetic trees indicated that three *Chpi*-IIB genes and two *Gaga*-IIB loci formed intra-species branches of the golden pheasant and chicken, respectively (Fig. 5B). Similarly, the *Chpi*-IAs and *Gaga*-IAs were grouped into the pheasant- and chicken-specific clusters, respectively (Fig. 5A). Therefore, these class I and II genes were orthologous in the golden pheasant and chicken. The corresponding reverse blocks of IIBs or IAs resulted from two inverted orthologous genes rather than inversion events. (2) The VISTA plots of multiple alignments revealed that the left and right non-coding spacers of the TAP2-TAP1 segment showed high identities between the golden pheasant and chicken, presenting a sharp contrast to blank plots induced by inversion (Fig. 4C). Similarly, the two flanking non-coding regions of TAPBP produced high homologies between these two birds, especially for the spacer linking TAPBP and IIB2 (Fig. 4C). Consequently, the TAPBP and TAP2-TAP1 were inverted alone.

The golden pheasant has two NK loci while chicken only one functional NK gene accompanied with a pseudo Blec gene [12] (Fig. 4B). The NK and C-type lection (i.e. Blec here) are both members of lection superfamily [33]. The dot plot indicated that the *Gaga*-Blec4 gene had obvious similarity to *Chpi*-NK1 and adjacent non-coding sequences (Fig. 4B). Futhermore, the VISTA plot showed that *Gaga*-Blec4 had perfect matching with the last exon and flanking sequences of *Chpi*-NK2. Hence, The *Gaga*-Blec4 should be a pseudo NK gene and thus pseudogenization of NK is characterized in chicken. Another difference in the number of genes is that chicken possesses two MHC class II beta genes but golden pheasant evolves three IIB loci. In combination with two functional NK genes, it seems that the golden pheasant potentially has better immunological function than chicken does.

### Phylogenetic relationships among several birds and inter-species structural variation in the MHC-B regions

Phylogenetic relationships of these four birds (golden pheasant, chicken, turkey and quail) have been demonstrated as ((golden pheasant, turkey) (chicken, quail)) based on mitogenomic data [34]. However, albeit that the NJ trees revealed that topologies of the genes IA, NK, DMB1 were in agreement with above-mentioned evolutionary relationships, the pairing between chicken and quail was supported by bootstrap values of 88% (IA), 43% (NK) and 74% (DMB1), respectively (Fig. 5). Conversely, the IIB loci grouped the chicken sequences into the cluster of golden pheasant and turkey with 99% bootstrap values (Fig. 5B). Furthermore, other genes like Blec, DMB2, TAP1 and TAP2 all classified chicken into the branch of turkey and golden pheasant showing bootstrap values as 65%, 69%, 49% and 75%, respectively (Fig. 5). As a consequence, it is manifest that the golden pheasant and turkey are sister groups, which were nearly all highly-supportive, but the pairing of chicken and quail is worthy of further consideration.

The VISTA plots indicated that the inversion of TAPBP was also observed in turkey but absent in both chicken and quail (Fig. 4C), suggesting that TAPBP might be inversed before the split between the ancestor of golden pheasant and turkey and other birds. The TAPBP-BRD2 segment showed varied genomic structures among these four species of *Galliformes* (Fig. 4C); chicken has only one IIB locus in this region [12], quail possesses one to three IIB loci in different haplotypes [32], both golden pheasant and turkey contain two class II B genes. Hosomichi *et al* [32] reported that there were more repeats and rearrangement elements in this region of quail in comparison with chicken, which could account for large variation in MHC genomic structure of quail.

The full sequencing of a 97-kb reverse-4D BAC demonstrated that the golden pheasant MHC-B locus contained 20 MHC-related genes and showed good synteny with that of chicken. The notable differences between these two species were the numbers of class II B loci and NK genes and the inversions of TAPBP gene and TAP1-TAP2 region. The phylogenetic trees supported that the golden pheasant and turkey has a common ancestor compared with chicken and quail. Hence, the shared inverted TAPBP was attributed to the inversion event happened before the split between the ancestor of golden pheasant and turkey and other birds, while the inverse TAP2-TAP1 was unique in the golden pheasant. The MHC data from the golden pheasant was reported for the first time, which would contribute to a better understanding of comparative genomics of the avian MHC.

## Materials and Methods

### Ethics statement

No blood samples were collected specifically for the purpose of research. The blood sample for BAC library was provided to us by zoo stuff when the bird was injured, and other five samples in this study were gifted by the government (Management Office of Tangjiahe National Nature Reserve) after the birds were rescued from poachers. As such, no ethics statement is required.

### Isolation of genomic DNA

The whole blood samples of five birds were used to isolate the genomic DNA using standard phenol-chloroform method [35]. The plasmid DNA of the pMD18-T vector clones and BACs were both isolated with an Axyprep plasmid miniprep kit (Axygen Biosciences).

### Pre-construction of a routine BAC library

We first constructed a mini routine BAC library in order to evaluate the genomic coverage of our new library without gridding clones according to a previous protocol [36]. Peripheral blood was obtained from a male golden pheasant, which was gifted by Hangzhou zoo. The whole blood was suspended in ice-cold phosphate-buffered saline (PBS) and the lymphocytes cells were harvested by centrifugation at 4,000 rpm for 4 min. The cells were resuspended in PBS reaching the concentration of 1×10^8^ cells/ml and mixed with an equal volume of liquefied (50°C) 2% certified low melt agarose (Bio-Rad, Hercules, USA). The whole mixture was poured into plug molds (Bio-Rad, Hercules, USA) and the obtained plugs were treated as described by Zeng *et al.* [37]. We performed pre-electrophoresis to remove inhibitors and small presumably sheared DNA molecules from the plugs and digested the DNA plugs in different *Hin*d III concentrations in order to determine the optimal conditions providing the largest number of fragments between 100 to 500 kb. After partial digestion, the DNA fragments 180 to 230 kb in size were recovered and ligated into the PCC1BAC vector (Epicentre, Madison, USA). After desalinization, the ligation products were transformed into Electrocompetent *E.coli* cells (Epicentre, Madison, USA). After incubation of one hour, the cultures (100 µl) were transferred to 10-cm plates and the clones were gridded into 96-well plates, thus obtaining a mini routine BAC library, which would be subjected to assess the rate of non-insert clones and the average size of inserts.

### Formal construction of a new BAC library

Once the parameters of the mini routine BAC library satisfy the standards, i.e. empty vector rate lower than 5% and average insert size larger than 100 kb, we will build a new BAC library skipping the procedure of gridding clones using the optimized conditions. After 1 h incubation above mentioned, the 100 µl cells cultures were added in 6 ml LB and grown at 37°C for 12–14 h. After full blending, the 6 ml LB culture was divided into three equal parts; the first one (part-I) is used to store the BAC library, the second one (part-II) is to isolate plasmid DNA for PCR, the third one (part-III) is to be cultured on the plates for picking clones into 96-well plates. Namely, large-size insets were not stored in single clones but in culture mixture in our modified BAC library. If the part-II shows a positive PCR test, the third copy would be subject to plate culture and clone picking, otherwise it will be storage as part-I for future.

### Insert size analysis

To analyze the size of insert DNA fragments in the library, 128 clones were randomly selected from the mini routine BAC library. The clones were cultured in 2 ml of LB medium containing 12.5 µg/ml chloramphenicol at 37°C under gentle agitation (280 rpm) for 10 h, and the DNA was isolated by alkaline lysis [38]. The DNA was electrophoresed through 0.8% agarose at 4°C for 3 hours using 4.0 V/cm. BAC-Tracker™ Supercoiled DNA ladder (New England Biolabs) was used as DNA size marker. The gel was stained with ethidium bromide and photographed. The size of BAC was calculated by Quantity One (BIO-RAD). The BAC end sequences were generated using the T7 (5′-TAA TAC GACTCA CTA TAG-3′) and RP-2 (5′-TAC GCC AAG CTA TTT AGG TGA GA-3′) vector primers. And the coverage of the library was estimated according to the formula W = NI/GS [39].

### Primer design, PCR amplification and sequencing

Based on the published sequences of chicken, turkey and quail, we designed several sets of primers of the B locus using Primer Primer [40], including IIB, DMB1, BRD, TAP1, and IA (Table 2). We employed three pairs of primers (IIB, BRD, and DMB1) to locate the multi-positive sub-libraries in order to exclude false positive. In total, we obtained 4, 5, 8 positive sub-libraries for the primers of IIB, BRD and DMB1, respectively, and achieved a multi-positive sub-library as No. 9-6 (Table 2). We performed primary screening on the clones in 96-well plates of No. 9-6 sub-library using the IIB primers and then verified the true positive BACs using multiple primer pairs (BRD, DMB1, IA, and TAP).

**Table 2:** Primer list for screening positive clones and verifying gene location.

Gene name	Location	Length (bp)	Tm (°C)	Primer sequence (5′ to 3′)	Positive sub-library^a^
IIB	Exon 2	221	60	Up: GGTGCGATCTTTGACTGCCAC	8-3, 9-1, **9-6**, 10-8
				Dn: TAGTTGTGCCGGCAGTAAGT	
BRD2	Exon 5–6	478	62	Up: AAGCGGAAAGCAGACACCACCAC	4-2, 6-5, **9-6**, 9-8, 9-9
				Dn: GGTAGTCCCGGTTCTCCATCTTC	
DMB1	Exon 3	180	58	Up: CTGCCAYGTKTGGGGCTTCT	6-5, 8-5, 8-7, 9-2, 9-4, **9-6**, 9-8, 9-9
				Dn: GMRTGCYRCACYGARCAYGTGTA	
TAP1	Exon 5–8	1445	59	Up: TTCCTCCTCTACCAGATACAGTTCAC	
				Dn: CAAAGAGCAGYGGCTCCTG	
I A	Exon 4	169	60	Up: GAGGTGCGAGTGTGGGGGAAGGAG	
				Dn: TGTGGTAGGTGCCGTCGCTGTTGG	
C3-Brd	IIB3-BRD2	4900	68 (two-step PCR)	Up: AGGGCCAATGAGCAGCAGGGTTG	
				Dn: CTGCCCGGCCCCTGTACATAAAATC	
Tpn-A6	TAPBP-IIB2	3390	68 (two-step PCR)	Up: GGGAGGGAACTGGGGGCAAACTGA	
				Dn: AGGGGGCTCTGGGGAAAGGATGGT	
DMB-TAP2	DMB2-TAP2	7621	68 (two-step PCR)	Up: TTGCAGAGGACATCCCACGAGGTA	
				Dn: TGTCGCTGCAAGGAAAGTCTACGA	
TAP1-C4	TAP1-C4	8449	68 (two-step PCR)	Up: GTGCCCAGGTCCTGCTCCGCTATT	
				Dn: ATGGGAAATGGGGCGTGGTTATGA	
Up4/Dn4	IIB2-IIB3	4186	68 (two-step PCR)	Up: GTGAACGGCACCCAGCAG	
				Dn: CCCGTAGTTGTGCCGGCAGTAC	

a The multi-positive sub-library is shown in bold.

The BACs indicating multiple positive PCR results were subject to end-sequencing and detection of insert size, of which the best one (No. 9-6-S2) was selected to commercial full BAC sequencing by shotgun method and performed on an ABI3730xl automated sequencer. Additionally, we designed inter-gene primers for the TAPBP-class II B loci, DMB-TAP and TAP-C4 in order to verify their locations by long range PCR (LR-PCR) amplification and sequence analysis in five individuals.

Common PCR reactions were performed using the genomic DNA of the individual from Hangzhou zoo. PCR reaction (40 µl total volume) included approximately 50 ng genomic DNA, 1.5 mM MgCl_2_, 25 pmol each primer, 100 uM dNTPs, and 1 U Taq polymerase (Takara). Amplifications were performed with the following steps: 5 min at 94°C; 35 cycles of 30 s at 94°C, 30 s at annealing temperature (Table 2), 30 s at 72°C, and a final extension of 5 min at 72°C. The program for LR-PCR was followed: 5 min at 94°C; 35 cycles of 40 s at 94°C, 4 min at 68°C, and a final extension of 5 min at 72°C. LaTaq (Takara) was used in LR-PCR of five individuals according to manufacturer instruction. PCR products were purified using an Axyprep PCR purification kit (Axygen Biosciences), ligated into the pMD18-T vector (Takara), and transformed into *Escherichia coli* DH5α competent cells. The positive clones were chosen for sequencing on an ABI 3730 automated sequencer, and six clones were picked in each PCR result of each individual.

### Sequencing, gene identification, annotation and phylogenetic analysis

The sequence of BAC was assembled with Phred [41]. The coding regions were predicted using GENESCAN ([42]; http://genes.mit.edu/GENSCAN.html). BLAST ([43]; www.ncbi.nlm.nih.gov/blast) was used for homology searches. The sequences of chicken were used as references. Repetitive elements and tRNAs elements were identified by REPEATMASKER (http://repeatmasker.org) and tRNAScan ([44]; http://lowelab.ucsc.edu/tRNAscan-SE/), respectively. CpG islands were elicited with Softberry CpGfinder (http://linux1.softberry.com/all.html), and GC content analysis was performed with 200-bp windows using Isochore ([45]; www.ebi.ac.uk/Tools/emboss/cpgplot/index.html). Sockeye [46] was employed to describe gene location. Dot matrix analysis was conducted by Pipmaker ([47]; http://bio.cse.psu.edu/pipmaker) and LBDot [48]. The program mVISTA ([49]; http://genome.lbl.gov/vista) was taken to do the multiple genomic aligments among the MHC-B sequences of chicken (AB268588; *Gaga-MHC*: BG1-TNXB), turkey (DQ993255; *Mega-MHC*: BG3-CenpA) and quail (AB078884; *Coja-MHC*: BG4-I E), respectively. The rates of nonsynonymous (*d_N_*) and synonymous (*d_S_*) nucleotide substitutions were calculated in MEGA 5 [50] according to the method of Nei and Gojobori with the Jukes-Cantor correction for multiple substitutions [51]. MEGA 5 [50] was also used to align multiple sequences, search the best evolutionary models, and construct phylogenetic trees using the Neighbor-Joining (NJ) method [52]. The bootstrap levels are calculated based on 1,000 replications.
